# Integrated Sensors Based on Low-Temperature Co-Fired Ceramic Technology for the Inside Pressure and Temperature Monitoring of Lithium-Ion Batteries

**DOI:** 10.3390/s25072095

**Published:** 2025-03-27

**Authors:** Wanjia Han, Mingsheng Ma, Yitong Guo, Zexi Yang, Zeyan Liu, Feng Liu, Jingjing Feng, Faqiang Zhang, Yingchun Lyu, Shigang Lu, Yongxiang Li, Jianjiang Bian, Zhifu Liu

**Affiliations:** 1 School of Materials Science and Engineering, Shanghai University, Shanghai 200444, China; hanwanjia@shu.edu.cn (W.H.);; 2 State Key Laboratory of High-Performance Ceramics and Superfine Microstructures, Shanghai Institute of Ceramics, Chinese Academy of Sciences, Shanghai 201899, China; 3 College of Sciences, Shanghai University, Shanghai 200444, China; 4 Materials Genome Institute, Shanghai University, Shanghai 200444, China; 5 School of Information Science and Technology, Shanghai Tech University, Shanghai 201210, China; 6 School of Engineering, RMIT University, Melbourne, VIC 3000, Australia

**Keywords:** battery monitoring, LTCC, integrated sensors, pressure, temperature

## Abstract

Monitoring internal pressure and temperature in lithium-ion batteries is essential for investigating internal chemical reactions, failure mechanisms, and providing early warnings of thermal runaway. The existing sensors face challenges in withstanding the high temperatures and corrosive electrolytes inside lithium-ion batteries. This work develops an integrated sensor with high robustness using low-temperature co-fired ceramic (LTCC) technology, which incorporates a multilayer ceramic circuit board, a digital pulse temperature sensor, a MEMS pressure sensor, and a microcontroller. It offers the real-time monitoring of pressure and temperature with digital output and calibrated accuracy, achieving a pressure resolution of 1 kPa with 0.085% F.S. accuracy and a temperature resolution of 0.1 °C with deviations under 0.5 °C. The pressure and temperature signals are independently output with drift below 0.067 kPa/°C. The integrated sensors were implanted into a pouch and prototype lithium-ion battery, respectively, for charge–discharge cycle monitoring. The results demonstrated that the integrated sensors could detect cyclic variations in pressure and temperature during charging and discharging until battery failure. Furthermore, the integrated sensors showed high stability after being immersed 60 days in the corrosive electrolyte, suggesting their potential as a novel method for monitoring the internal pressure and temperature of lithium-ion batteries.

## 1. Introduction

Due to their high energy density, long cycle life, and low cost, lithium-ion batteries are widely used in electric vehicles, energy storage systems, and various portable electronic devices today [1,2]. However, thermal runaway combustion accidents of lithium-ion batteries may occur at any time due to internal short circuits and flammable liquid electrolytes. One of the most effective ways to prevent thermal runaway is to monitor the real-time status of lithium-ion batteries using various sensors. [3]. The widely used battery management systems (BMS) primarily monitor the current, voltage, and temperature of the battery pack externally. They can also provide the battery’s state of charge (SOC), state of health (SOH), and structural changes. When alarm thresholds are triggered, the response time left for intervention is very limited. In contrast, monitoring the internal status parameters of a battery provides more precise insights into the battery’s electrochemical and mechanical changes, enabling much earlier warnings and intervention [4,5,6,7]. The electrochemical reactions during battery cycling can lead to significant fluctuations in the internal temperature of the battery [8,9]. In addition, electrolyte decomposition and side reactions at the electrodes inevitably generate gases, such as carbon dioxide, carbon monoxide, methane, and hydrogen [10,11,12]. The accumulation of these gases inside the battery cell increases the internal gas pressure and volume, which may lead to cell rupture, leakage, or even explosion. Therefore, monitoring internal temperature and pressure is crucial for the early detection of thermal runaway and for assessing the battery’s health.

The reported implantable sensors for the internal status monitoring of batteries include thin-film sensors based on organic substrates and optical fiber sensors utilizing the diffraction principle [13]. Zhu et al. [14] fabricated flexible temperature and strain sensors resistant to internal battery corrosion by depositing platinum metal electrodes on polyimide thin films. Thin-film sensors offer advantages, such as small size, minimal impact on the battery during implantation, and the use of electrical signals for sensing, which facilitates integration with conditioning circuits. However, the organic substrates used in these sensors face challenges in enduring high temperatures exceeding 400 °C during thermal runaway. Additionally, challenges remain in decoupling multiple signals, including temperature, pressure, and strain. Gervillié et al. [15] developed an infrared spectroscopy technology based on chalcogenide optical fibers and successfully applied it to commercial 18650 sodium-ion/lithium-ion batteries. Mei et al. [16] developed a multifunctional optical fiber sensor with a diameter of 125 μm, by incorporating Bragg gratings into optical fibers. These optical fiber sensors exhibit high-temperature and corrosion resistance, and their ability to incorporate multiple sensing points on a single fiber reduces the need for additional wiring. However, these sensors require more complex signal modulation and demodulation systems, making them less suitable for real-time monitoring in applications such as electric vehicles and portable devices.

Low-temperature co-fired ceramics (LTCC) allow for the co-firing of ceramics with highly conductive Au, Ag, and Cu metal electrodes, enabling multilayer wiring and interlayer via metal interconnections. LTCC offers advantages, such as multilayer high-density wiring, integrates passive components with the substrate, and provides self-packaging, which enhances the sensor’s integration and reliability [17]. The electrical, mechanical, and thermal properties of LTCC materials can be flexibly adjusted through formulation, and they can also accommodate complex structural designs, such as irregular shapes and cavities, offering excellent design flexibility. These properties make LTCC particularly suitable for fabricating integrated sensors for harsh environments. For instance, Fournier et al. [18] designed pressure and temperature sensors with integrated signal conditioning electronics for linearization, regulation, and temperature compensation and also developed an airflow sensor. Lin et al. [19] developed a passive wireless pressure/temperature dual-parameter LC resonant sensor by creating cavities and electrodes in the LTCC substrate. This sensor achieved a pressure range of 140–850 kPa and a temperature range of 50–500 °C. These studies demonstrate that LTCC can be used to create sensors that integrate information communication and status sensing, making it an ideal platform for integrated sensors designed to monitor the internal state of lithium-ion batteries. However, there are few reports on the research into the internal state monitoring of lithium-ion batteries using LTCC technology.

In this work, a novel integrated sensor consisting of an LTCC substrate, an IC temperature sensor, a MEMS pressure sensor, and controlling elements is proposed. The feasibility of using this sensor for the internal pressure and temperature monitoring of lithium-ion batteries is also demonstrated.

## 2. Experiment

### 2.1. Sensor Design

**Figure 1:**
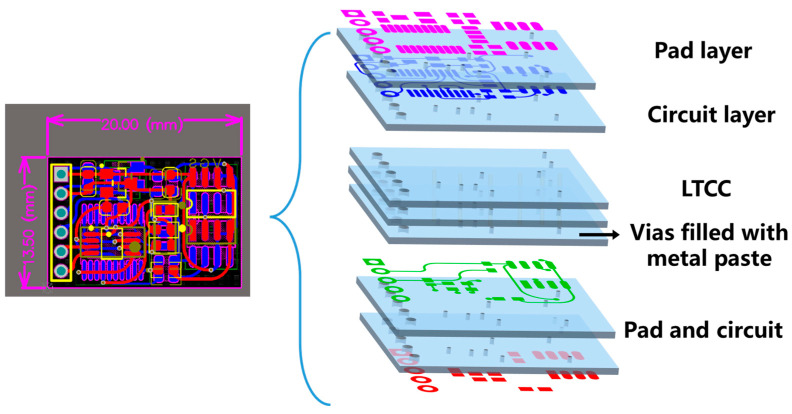
LTCC circuit substrate diagram and multilayer structure.

The integrated sensor comprises a multilayer ceramic circuit substrate, a temperature sensor, a pressure sensor with its signal conditioning chip, and a microcontroller unit (MCU). The temperature sensor is a digital pulse type (NST1001, Novosense, Suzhou, China), and the pressure sensor is a silicon piezoresistive type (LMSPAS8M150, Suzhou Lanmei Electronics Co., Ltd., Suzhou, China). Each sensor converts temperature and pressure variations into resistance changes, respectively. These resistance changes are then converted into analog voltage signals by the conditioning chip and transmitted to the microcontroller (AT32F421F8P7, ARTERY, Chongqing, China). The multilayer circuit layout is shown in Figure 1.

### 2.2. Sensor Preparation

**Figure 2:**
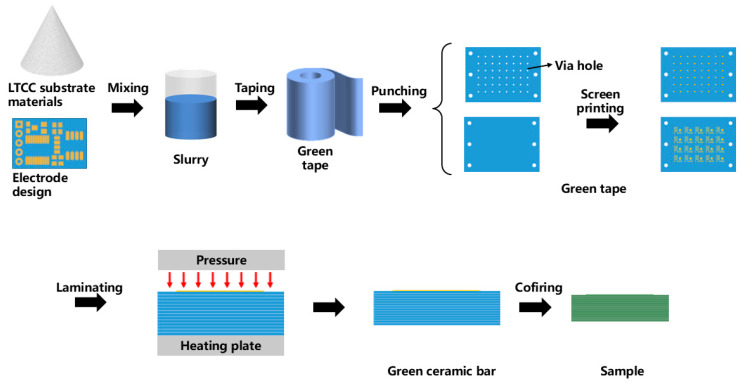
LTCC preparation process for a multilayer circuit substrate.

The sensor fabrication process primarily involves the following two steps: the preparation of the multilayer ceramic substrate and the soldering assembly of the surface-mount components. The multilayer ceramic substrate is fabricated using the LTCC process, as shown in Figure 2. The LTCC substrate material and the silver paste are prepared by the Siramic-Tech Co., Ltd., Jinhua, China. Each single-layer green tape has a thickness of 130 µm.

**Figure 3:**
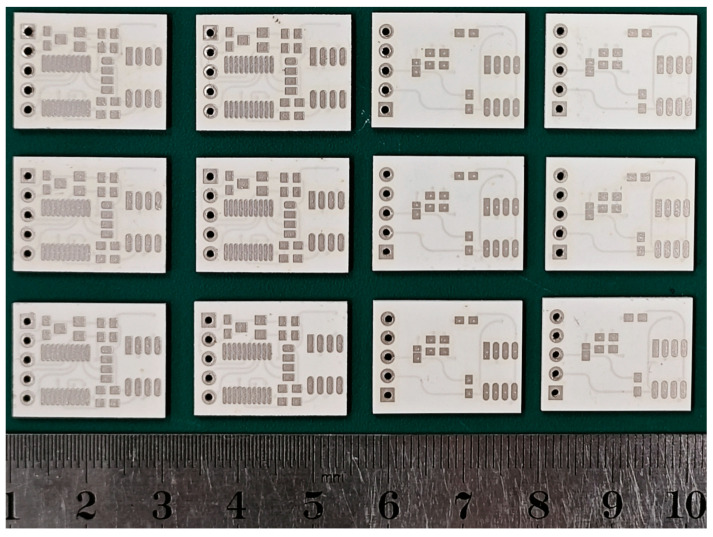
Sintered multilayer ceramic circuit substrate.

In accordance with the multilayer ceramic circuit design shown in Figure 1, the green tapes are cut and punched, and vias are filled with silver paste. The designed circuit and pad patterns are then printed onto the green tapes using screen printing, as illustrated in Figure 3. The 10 layers of green tape, with vias filled, are laminated at 65 °C and 15 MPa for 20 s to form a multilayer ceramic green bar. This green bar is then heated in a sintering furnace to 450 °C at a rate of 1.5 °C/min, with a two-hour hold to decompose and remove all organic binders. The temperature is then raised to 850 °C within 80 min and held for 30 min to complete the sintering process. The final dimensions of the obtained multilayer ceramic circuit substrate are 20 mm × 13.5 mm × 1 mm.

**Figure 4:**
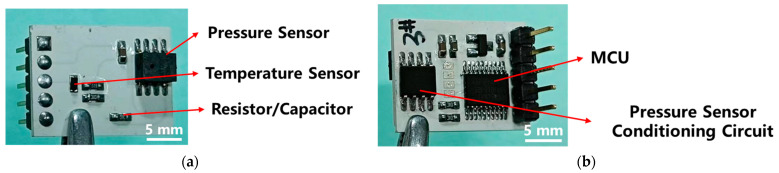
(**a**) Front side and (**b**) back side of the LTCC sensor.

The microcontroller, signal conditioning chip, pressure and temperature sensors, resistors, capacitors, and other components are then assembled through a reflow soldering process. Additionally, the pins or wiring for signal transmission are soldered to complete the integrated sensors. As shown in Figure 4, the integrated sensors feature a double-sided design, with the pressure and temperature sensors on the front side (Figure 4a) and the microcontroller and signal conditioning chip on the back side (Figure 4b). The metal electrode circuit is embedded within the ceramic substrate, thereby avoiding the risk of surface electrodes being affected by corrosive electrolyte immersion.

### 2.3. Sensor Calibration

**Figure 5:**
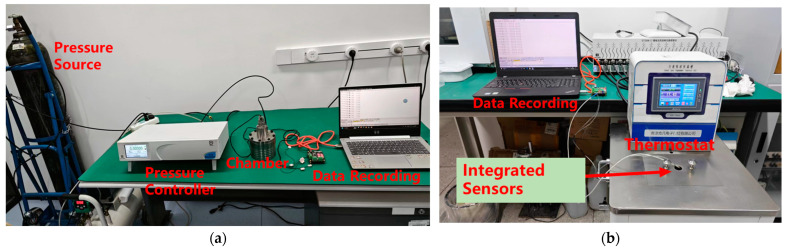
Sensor test apparatus: (**a**) pressure and (**b**) temperature.

For the pressure test, a standard air pressure controller (WIKA, CPC4000, Klingenberg, Germany) is used to generate a reference air pressure as the set value. The test setup is shown in Figure 5a. The sensor is placed in a test fixture consisting of a sealed gas chamber and an aviation connector. The aviation connector ensures both electrical connectivity and airtightness, while the sealed chamber maintains the stability of the set pressure. The pressure test range of the sensor is from 90 kPa to 1 MPa absolute pressure. This range covers the slight negative pressure inside the battery due to temperature fluctuations and the pressure increase caused by gas generation during battery operation. The test points are set to sample at intervals of 100 kPa. Each test point is held for 5 min to ensure that the set pressure and sensor readings are stable. During the test, the system is first pressurized at both the lower and upper pressure limits (0 kPa and full scale), followed by pressure cycling, according to the set step value. The process is repeated three times, and the data are recorded. The static performance is then calculated using the method outlined in Section 2.4, which includes parameters such as repeatability, non-linearity, accuracy, and hysteresis.

The temperature test setup is shown in Figure 5b. The temperature control is achieved using a precisely calibrated silicone oil thermostat (Nanjing Kenfan, KDC400, Nanjing, China). During the test, the sensor is fully immersed in the silicone oil, ensuring complete thermal contact with the oil for uniform temperature distribution. The sensor’s temperature test range is from −30 °C to 150 °C, with sampling points set at intervals of 10 °C. Each test point is held for 10 min to ensure that the set temperature and sensor readings are stable. The test is repeated three times, with data recorded and static performance calculated following the method outlined in Section 2.4.

The testing device for pressure–temperature independence testing uses the same sealed chamber and signal acquisition system as the pressure testing setup. During the test, the sealed chamber is placed into a thermostat, which is used for temperature testing, to provide varying temperature changes. The corresponding pressure output variations from the sensors are detected and recorded, from which the pressure–temperature independence can be calculated.

### 2.4. Method for Calculating Static Sensor Performance

Calculation of the average value of measurement data, characteristic equation, and measurement range:

Let there be m calibration points within the entire measurement range of the sensor, and n pressure/temperature cycling calibration tests are performed. At each calibration point, there are n sets of calibration data for both the forward and reverse cycles. The average value of the forward cycle test data at each calibration point is calculated using Equation (1), the average value of the reverse cycle test data is calculated using Equation (2), and the overall average value is calculated using Equation (3).

(1) $${\bar{Y}}_{U_{i}}=\frac{1}{n}\sum_{j=1}^{n}\;Y_{U_{ij}}$$

(2) $${\bar{Y}}_{D_{i}}=\frac{1}{n}\sum_{j=1}^{n}\;Y_{D_{ij}}$$

(3) $${\bar{Y}}_{i}=\frac{1}{2}\left({\bar{Y}}_{U_{i}}+{\bar{Y}}_{D_{i}}\right)$$

In the equations, $Y_{U_{ij}}$ represents the reading of the forward cycle at the i-th calibration point for the j-th test (where i = 1,2,3,…m and j = 1,2,3,…n); $Y_{D_{ij}}$ represents the reading of the reverse cycle at the i-th calibration point for the j-th test (where i = 1,2,3,…m and j = 1,2,3,…n); and n denotes the number of repeated tests.

The general form of the linear sensor characteristic equation is given by Equation (4), where intercept a and slope b are calculated using Equations (5) and (6), respectively. In the equations, $X_{i}$ represents the pressure value at the i-th identification point (where i = 1,2,3,…m); ${\bar{Y}}_{i}$ represents the total average value of the forward and reverse cycles at the i-th identification point; and m denotes the number of calibration points.

(4) Y=a+bX

(5) $$a=\frac{\sum_{i=1}^{m}X_{i}^{2}\sum_{i=1}^{m}{\bar{Y}}_{i}-\sum_{i=1}^{m}X_{i}\sum_{i=1}^{m}X_{i}{\bar{Y}}_{i}}{m\sum_{i=1}^{m}X_{i}^{2}-{\left(\sum_{i=1}^{m}X_{i}\right)}^{2}}$$

(6) $${\bar{Y}}_{i}=\frac{1}{2}\left({\bar{Y}}_{U_{i}}+{\bar{Y}}_{D_{i}}\right)$$

The full-scale output of the sensor is defined as the absolute value of the difference between the upper and lower limit output values (based on the calculated theoretical characteristic line) and is calculated using Equation (7). In the equations, b represents the slope of the theoretical characteristic line; $X_{H}$ and $X_{L}$ represent the pressure values at the upper and lower measurement limits, respectively.

(7) $$Y_{FS}=|b(X_{H}-X_{L})|$$

The non-linearity of the linear sensor is calculated using Equation (8), where ${\bar{Y}}_{i}$ is the total average value; $Y_{i}$ is the value calculated from linear sensor characteristic equation; and $Y_{FS}$ is the full-scale output calculated from Equation (7).

(8) $$\xi_{L}=\frac{|{\bar{Y}}_{i}-Y_{i}{|}_{max}}{Y_{FS}}\times 100\%$$

The hysteresis of the sensor is calculated using Equation (9), where ${\bar{Y}}_{U_{i}}$ and ${\bar{Y}}_{D_{i}}$ are the average values of the forward and reverse cycle readings, respectively, at the same calibration point; and $Y_{FS}$ is the full-scale output.

(9) $$\xi_{H}=\frac{|{\bar{Y}}_{U_{i}}-{\bar{Y}}_{D_{i}}{|}_{max}}{Y_{FS}}\times 100\%$$

The sub-sample standard deviation for both the forward and reverse cycles at each calibration point is calculated using the Bezier formula. The forward cycle sub-sample standard deviation is calculated using Equation (10), the reverse cycle sub-sample standard deviation is calculated using Equation (11), and the overall sub-sample standard deviation of the sensor across the entire measurement range is calculated using Equation (12), where $Y_{U_{ij}}$ is the reading at the i-th calibration point and j-th test of the forward cycle; $Y_{D_{ij}}$ is the reading at the i-th calibration point and j-th test of the reverse cycle; ${\bar{Y}}_{U_{i}}$ is the average value of the forward cycle at the i-th calibration point; ${\bar{Y}}_{D_{i}}$ is the average value of the reverse cycle at the i-th calibration point; n is the number of repeated tests; and n is the number of repeated tests.

(10) $$S_{U_{i}}=\sqrt{\frac{1}{n-1}\sum_{j=1}^{n}\;(Y_{U_{ij}}-{\bar{Y}}_{U_{i}}{)}^{2}}$$

(11) $$S_{D_{i}}=\sqrt{\frac{1}{n-1}\sum_{j=1}^{n}\;(Y_{D_{ij}}-{\bar{Y}}_{D_{i}}{)}^{2}}$$

(12) $$S=\sqrt{\frac{1}{2m}\left(\sum_{i=1}^{m}\;S_{U_{i}}^{2}+\sum_{i=1}^{m}\;S_{D_{i}}^{2}\right)}$$

The repeatability is calculated according to Equation (13), where λ is the coverage factor; S is the sub-sample standard deviation; and $Y_{FS}$ is the full-scale output.

(13) $$\xi_{R}=\frac{\lambda S}{Y_{FS}}\times 100\%$$

The accuracy of the sensor is a comprehensive reflection of the systematic and random errors, which depend on the magnitude of the systematic error band $U_{1}$ and the random error band $U_{2}$. The systematic error for the forward cycle is calculated using Equation (14), and the systematic error for the reverse cycle is calculated using Equation (15), where Y represents the ideal value. The systematic error band $U_{1}$ of the sensor is taken as the larger of $(\Delta Y{)}_{U_{i}}$ and ${\left(\Delta Y\right)}_{D_{i}}$. The random error band $U_{2}$ of the sensor is calculated according to Equation (16), where S represents the sub-sample standard deviation of the sensor across the entire measurement range.

(14) $$(\Delta Y{)}_{U_{i}}=|{\bar{Y}}_{U_{i}}-Y{|}_{max}$$

(15) $${\left(\Delta Y\right)}_{D_{i}}={\left|{\bar{Y}}_{D_{i}}-Y\right|}_{max}$$

(16) $$U_{2}=\pm 3S;$$

The accuracy of the sensor is then calculated using Equation (17).

(17) $$\xi =\pm \frac{\left|U_{1}|+|U_{2}\right|}{Y_{FS}}\times 100\%;$$

### 2.5. Sensor Test

**Figure 6:**
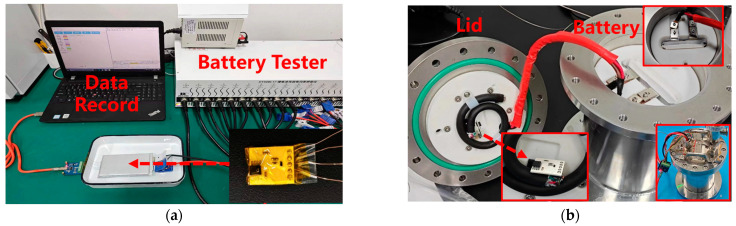
(**a**) Sensor implanted in a pouch battery; (**b**) sensor implanted in a prototype battery.

To verify whether the sensor can detect pressure/temperature changes during battery operation, as well as the sensor’s endurance to the internal environment of the battery, the sensor is implanted into a pouch-type lithium iron phosphate (LiFePO_4_) battery and a simulated cylindrical battery, respectively. The battery used has a capacity of 12 ampere-hours and dimensions of 120 mm × 80 mm × 10 mm. Figure 6a shows a photograph of the sensor implanted inside the pouch-type battery and undergoing a cycle charge–discharge test. The sensor is protected with polyimide tape for insulation. The implantation of the sensor is completed in an argon-protected glovebox, and the battery is sealed with adhesive. After the adhesive is fully cured, the sensor-implanted battery is connected to a lithium-ion battery charge–discharge tester (DEKANG DT50W-17, Hangzhou, China) for 100 charge–discharge cycles, with a charge/discharge rate of 0.5C and a rest period of 0.5 h between cycles. Simultaneously, the sensor is activated to collect and record internal pressure and temperature variations during the battery’s charge–discharge process.

Figure 6b shows a photograph of the sensor implanted inside a prototype battery undergoing charge–discharge cycle testing. The prototype battery consists of a metal canister with a sealing lid and an aviation plug on the lid, designed to simulate the structure of a cylindrical battery. During the test, a pouch-type battery identical to that in Figure 6a is used and cut open in an argon-protected glovebox, and the battery is placed inside the canister. The positive and negative terminals are connected via the aviation plug for charge–discharge testing. The sensor is implanted in the gap between the canister lid and the battery cell, with power and data transmission facilitated through the aviation plug. Once assembled, the canister and lid are sealed, and the battery undergoes 100 charge–discharge cycles with a charge rate of 0.6C and a discharge rate of 1C. Simultaneously, the sensor is activated to monitor and record the internal pressure and temperature variations during the charge–discharge process.

## 3. Results and Discussion

**Figure 7:**
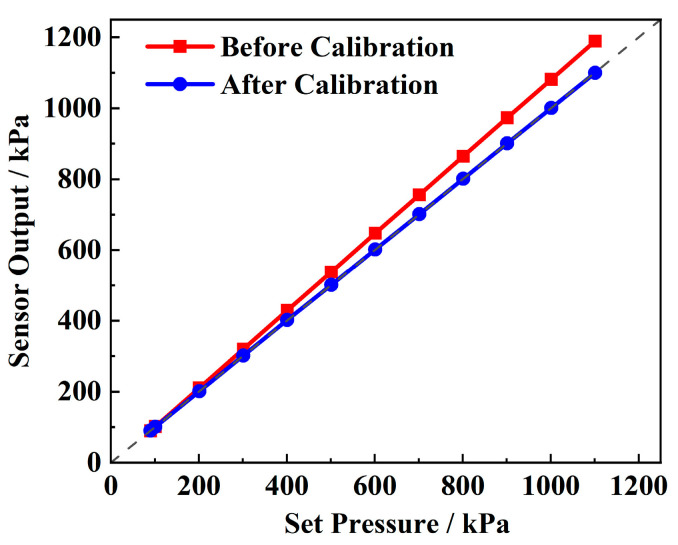
Pressure sensor calibration.

Figure 7 presents the static performance test curve of the pressure sensor and the sensor performance after linear calibration. It can be observed that, as the applied pressure increases, the deviation of the pressure sensor’s output becomes more significant, with the overall output exceeding the true value. The calculated overall error over the full range is 7.45%. The sensor exhibits excellent linearity and accuracy, with a non-linearity of 0.092%, a repeatability deviation of 0.067%, and a fitting R^2^ of 0.99. This allows for a significant improvement in accuracy through linear correction. Using the least squares fitting method, the calibration Equation (18) is derived, and the linear correction Equation (19) is subsequently obtained. After correction, the comprehensive error is reduced by two orders of magnitude, reaching ±0.085%, with a resolution of 1 kPa, demonstrating excellent pressure sensing performance.

(18) $$Y_{1}=-8.18788+1.08903X;$$

(19) $$Y_{2}=(Y_{1}+8.18788)/1.08903;$$

**Figure 8:**
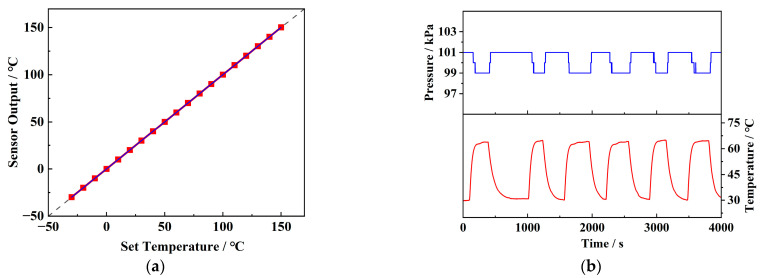
(**a**) Temperature sensor test and (**b**) temperature drift test of pressure sensors.

Figure 8a illustrates the static performance test curve for the temperature response of the sensor. It shows a high resolution of 0.1 °C within the range of −30 °C to 150 °C and a rapid response time of 0.21 s. The sensor also exhibits high accuracy, with a comprehensive error of ±0.15%, allowing for precise temperature measurement without additional correction. Figure 8b shows the temperature drift test curve of the pressure sensor, with an output attenuation of about 2 kPa, as the temperature rises from room temperature to 65 °C. This attenuation can be attributed to the increased lattice vibrations in silicon at higher temperatures, which reduce the modulation effect of stress on carrier mobility and decrease the piezoresistive coefficient [20]. Nevertheless, the sensor exhibits excellent repeatability during multiple heating and cooling cycles, allowing for temperature drift correction through test data, thus maintaining good accuracy over a wide temperature range. Since the internal pressure and temperature often change simultaneously during battery charge and discharge cycles, it is crucial for the pressure sensor to maintain accuracy under temperature variations. Table 1 summarizes the static performance of the sensor after correction.

In addition, due to the enclosed environment inside the battery, gas expansion caused by temperature increases can lead to pressure variations, which interfere with the monitoring of internal pressure changes. Therefore, correction for this effect is also required. The internal environment of the battery is generally argon, which can be approximated as an ideal gas, and the internal state can be described by the Ideal Gas Law (20).

(20) pV=nRT

**Figure 9:**
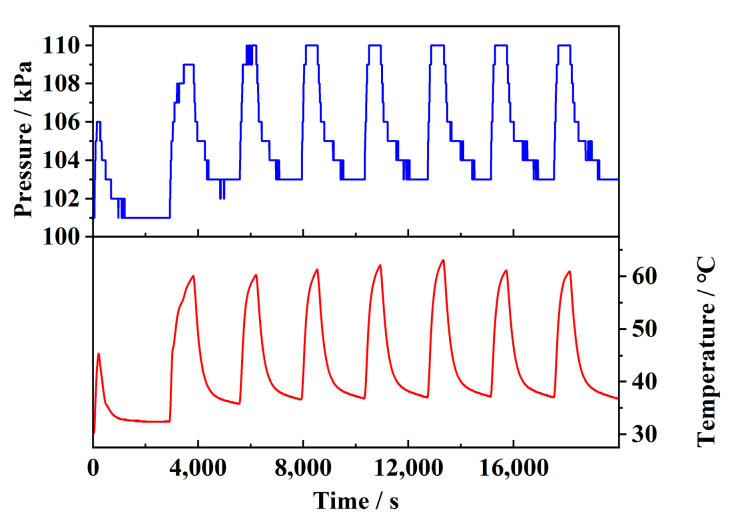
Gas expansion caused by temperature changes.

The correction factor is determined experimentally. Figure 9 illustrates the pressure variation caused by temperature changes in a closed environment, with seven cycles performed. The relationship between pressure and temperature was analyzed using the method of linear fitting. The result shows that the data approximately follow a linear trend, with an R^2^ value of 0.95, consistent with Equation (20).

Based on the experimental data, the correction equation was determined as Equation (21). By considering both temperature drift and gas expansion effects, the pressure readings from the sensor can be corrected using Equation (22), effectively eliminating interference. In the equation, $P_{i}$ represents the sensor’s reading, while $P_{0}$ denotes the corrected true value.

(21) p=0.31779T(°C)+91.13592

(22) $$P_{0}=P_{i}-0.130325(T_{i}-25\;{}^{\circ}C)$$

**Figure 10:**
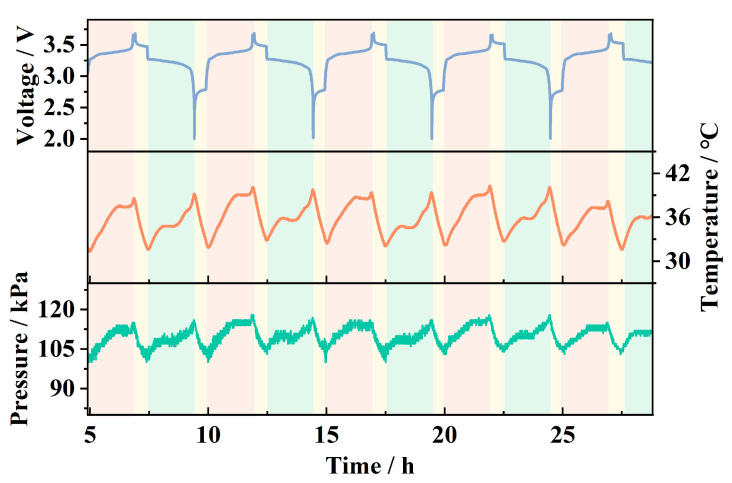
Internal pressure and temperature variations with voltage.

Figure 10 shows the internal pressure and temperature data collected by the sensor during the charge–discharge cycle of the pouch battery, along with the linear correction and temperature drift compensation applied to the pressure data, as described above. It can be observed that, as the battery voltage periodically increases and decreases, the sensor detects corresponding periodic variations in pressure and temperature. The temperature fluctuates between approximately 30 °C and 40 °C, while the pressure varies between around 102 kPa and 120 kPa. The periodic temperature rise is primarily attributed to the Joule heating effect caused by the current, while the temperature drop occurs during the rest periods between charge and discharge cycles. The periodic pressure changes are a result of reversible strain in the battery cell due to the adsorption and desorption of lithium ions in the electrode material [21,22]. During charging, lithium ions migrate through the electrolyte to the anode, embedding into the anode material and causing it to expand. The expanded electrode compresses the air inside the pouch battery, leading to an increase in pressure detected by the sensor. During discharging, the lithium ions detach from the anode material, causing the battery strain to recover, which leads to a decrease in pressure.

**Figure 11:**
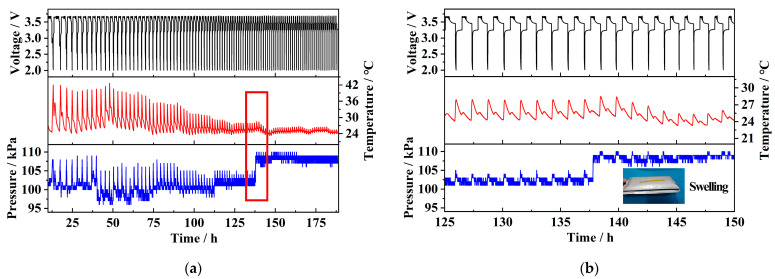
(**a**) 100-cycle charge/discharge of pouch cell; (**b**) key point of battery failure and swelling.

Figure 11a shows the data curve of the pouch battery after 100 charge–discharge cycles. It can be observed that the time required to complete one full charge–discharge cycle gradually decreases, while the amplitude of the corresponding temperature and pressure changes also decreases. This indicates that the battery is gradually degrading, exhibiting significant capacity loss. After 100 cycles, the battery experienced swelling. As shown in the red square in Figure 11a, which is then magnified in Figure 11b, approximately 140 h after the test began, the sensor detected a temperature peak and an almost simultaneous pressure step change. Following this point, the amplitude of temperature and pressure variations remained almost unchanged, with the pressure value permanently increasing by approximately 5 kPa. It can be concluded that this sudden temperature and pressure change marks the critical point of the battery’s total failure and swelling. After this point, the battery was permanently damaged, making it unable to perform normal charge–discharge cycles. This was reflected in rapid voltage fluctuations with an almost constant temperature and pressure. The entry of moisture through the gap left in the battery during sealing, as well as the volatilization of the electrolyte inside the battery, has been identified as the cause of battery failure and swelling. This leads to the growth of lithium dendrites and side reactions in the solid electrolyte interphase (SEI) layer, resulting in its thickening or rupture, which in turn, causes electrode material degradation and electrolyte decomposition [23].

**Figure 12:**
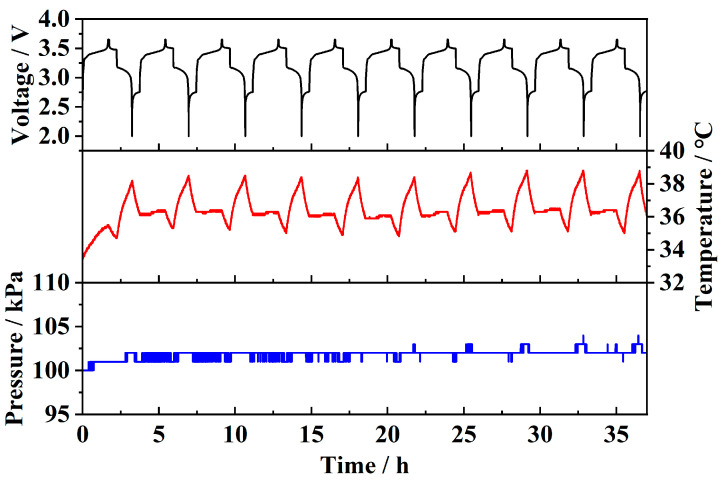
Prototype battery monitoring results.

Figure 12 shows the test results of the sensor embedded in a prototype battery. It can be observed that the temperature fluctuates periodically during charge–discharge cycles. Since the discharge rate (1C) is higher than the charge rate (0.6C), and charging power is proportional to the square of the current, the temperature rise during discharge is significantly higher than during charging. Due to the large internal space and high air content of the prototype battery, and the fact that the sensor is integrated into the lid, it cannot directly measure reversible strain during charge–discharge cycles. As a result, only minimal pressure fluctuations (around 2 kPa) can be detected. These pressure fluctuations are primarily due to strain-induced pressure changes and a small amount of gas generated by side reactions. This demonstrates that the sensor can monitor temperature, strain, and pressure parameters during battery operation in both pouch and prototype batteries and can also detect changes in physical parameters associated with battery failure, serving as a basis for fault diagnosis.

After completing 100 cycles of battery monitoring, the sensor was left inside the battery for a total implantation time of over 60 days. The sensor was cleaned with alcohol, and its surface and solder joints were examined under a microscope. After two months of service in the corrosive environment inside the battery, no significant corrosion was observed on the ceramic surface or the metal solder joints of the sensor. The sensor was then subjected to a static performance test, shown in Table 2. The results indicated that, after long-term implantation in the battery, the sensor performance exhibited no significant drift or degradation, demonstrating its excellent resistance to chemical corrosion.

In summary, there are mainly two kinds of implantable sensors used for monitoring the internal state of the lithium-ion batteries in the previous reports: the flexible sensors based on organic thin films and the fiber-optic sensors. Most of the reported sensors focused on measuring the internal temperature and strain during the charge–discharge process of lithium-ion batteries. It is worth noting that the internal gas pressure variations in the batteries are also considered to be a critical indicator of battery status, which are closely related to lithium-ion intercalation and deintercalation in electrode materials, abnormal side reactions, and gas generation [8]. However, the implantable sensors for internal gas pressure monitoring are still very rare. On the other hand, the flexible sensors based on organic thin films have a very limited working temperature, making them unsuitable for withstanding high temperatures up to above 500 °C during the lithium battery thermal runaway [16]. As a new sensor solution for the internal hash environment in the battery, the feasibility of the LTCC-integrated sensor for monitoring the internal temperature and pressure in the lithium battery was demonstrated for the first time in this study. It offers much valuable gas pressure monitoring data, which can serve as important reference for battery internal electrochemical mechanism studies. Furthermore, the results in this study also provided some very useful information for the sensor and battery researchers, such as the integration of the sensor elements and circuits in the LTCC substrate, the sensor calibration methods, and the law of the temperature and pressure parameter variation in the internal in situ state of the battery.

## 4. Conclusions

This study proposes an integrated sensor based on LTCC technology, which is suitable for internal state monitoring of lithium-ion batteries. The pressure resolution reaches 1 kPa, with an accuracy of 0.085% F.S., and the temperature resolution is 0.1 °C, with a deviation of 0.15%. The pressure and temperature signals are independently output with a drift of less than 0.067 kPa/°C, and they can be corrected. The sensor can track the periodic variations in pressure and temperature during charge–discharge cycles, as well as detecting critical temperature peaks and pressure steps when the battery shows signs of failure and swelling. After 100 charge–discharge cycles and 60 days of implantation, the sensor shows no significant drift in performance, and no obvious corrosion is observed. The sensor demonstrates high reliability, compact size, and high integration, making it a feasible method for battery state monitoring research. It also shows potential in fields such as smart battery systems, IoT sensing, and corrosion/harsh environmental sensing.

In future research, the efforts will be made to obtain multi-parameter integration sensors with pressure, temperature, and gas using LTCC technology and integrate them with signal circuits and battery cells. Moreover, continued exploration of using LTCC sensors for internal battery monitoring will be pursued, with the aim of providing a new technological solution for monitoring the internal state of batteries.

## Figures and Tables

**Table 1:** Static performance of the integrated sensor.

	Non-Linearity	Hysteresis	Repeatability	Accuracy	Sensitivity
**Temperature**	0.080%	0.13%	0.16%	0.15%	0.1 °C
**Pressure**	0.092%	0.090%	0.067%	0.085%	1 kPa
**Drift**			<0.067 kPa/°C		

**Table 2:** Sensor static performance after 60 days of battery implantation.

		Non-Linearity	Hysteresis	Repeatability	Accuracy	Sensitivity
**Before**	**Temperature**	0.080%	0.13%	0.16%	0.15%	0.1 °C
	**Pressure**	0.092%	0.090%	0.067%	0.085%	1 kPa
**After**	**Temperature**	0.080%	0.13%	0.16%	0.15%	0.1 °C
	**Pressure**	0.092%	0.091%	0.068%	0.090%	1 kPa

## Data Availability

Data are contained within the article.
